# Antibiofilm Activity of 3D-Printed Nanocomposite Resin: Impact of ZrO_2_ Nanoparticles

**DOI:** 10.3390/nano13030591

**Published:** 2023-02-01

**Authors:** Abdulrahman Khattar, Jawad A. Alghafli, Mohammed A. Muheef, Ali M. Alsalem, Mohammed A. Al-Dubays, Hussain M. AlHussain, Hussain M. AlShoalah, Soban Q. Khan, Doaa M. AlEraky, Mohammed M. Gad

**Affiliations:** 1 College of Dentistry, Imam Abdulrahman Bin Faisal University, P.O. Box 1982, Dammam 31441, Saudi Arabia; 2 Department of Dental Education, College of Dentistry, Imam Abdulrahman Bin Faisal University, P.O. Box 1982, Dammam 31441, Saudi Arabia; 3 Department of Biomedical Dental Sciences, College of Dentistry, Imam Abdulrahman Bin Faisal University, P.O. Box 1982, Dammam 31441, Saudi Arabia; 4 Department of Substitutive Dental Sciences, College of Dentistry, Imam Abdulrahman Bin Faisal University, P.O. Box 1982, Dammam 31441, Saudi Arabia

**Keywords:** 3D printing, ZrO_2_ nanoparticles, denture base, *Candida albicans*, cell proliferation assay

## Abstract

Poly(methyl methacrylate) (PMMA) is a commonly used material, as it is biocompatible and relatively cheap. However, its mechanical properties and weak antibiofilm activity are major concerns. With the development of new technology, 3D-printed resins are emerging as replacements for PMMA. Few studies have investigated the antibiofilm activity of 3D-printed resins. Therefore, this study aimed to investigate the antibiofilm activity and surface roughness of a 3D-printed denture base resin modified with different concentrations of zirconium dioxide nanoparticles (ZrO_2_ NPs). A total of 60 resin disc specimens (15 × 2 mm) were fabricated and divided into six groups (n = 10). The groups comprised a heat-polymerized resin (PMMA) group, an unmodified 3D-printed resin (NextDent) group, and four 3D-printed resin groups that were modified with ZrO_2_ NPs at various concentrations (0.5 wt%, 1 wt%, 3 wt%, and 5 wt%). All specimens were polished using a conventional method and then placed in a thermocycler machine for 5000 cycles. Surface roughness (Ra, µm) was measured using a non-contact profilometer. The adhesion of *Candida albicans* (*C. albicans*) was measured using a fungal adhesion assay that consisted of a colony forming unit assay and a cell proliferation assay. The data were analyzed using Shapiro–Wilk and Kruskal–Wallis tests. A Mann–Whitney *U* test was used for pairwise comparison, and *p*-values of less than 0.05 were considered statistically significant. The lowest Ra value (0.88 ± 0.087 µm) was recorded for the PMMA group. In comparison to the PMMA group, the 3% ZrO_2_ NPs 3D-printed group showed a significant increase in Ra (*p* < 0.025). For the 3D-printed resins, significant differences were found between the groups with 0% vs. 3% ZrO_2_ NPs and 3% vs. 5% ZrO_2_ NPs (*p* < 0.025). The highest Ra value (0.96 ± 0.06 µm) was recorded for the 3% ZrO_2_ NPs group, and the lowest Ra values (0.91 ± 0.03 µm) were recorded for the 0.5% and 5% ZrO_2_ NPs groups. In terms of antifungal activity, the cell proliferation assay showed a significant decrease in the *C. albicans* count for the 0.5% ZrO_2_ NPs group when compared with PMMA and all other groups of 3D-printed resins. The group with the lowest concentration of ZrO_2_ NPs (0.5%) showed the lowest level of *C. albicans* adhesion of all the tested groups and showed the lowest *Candida* count (0.29 ± 0.03). The addition of ZrO_2_ NPs in low concentrations did not affect the surface roughness of the 3D-printed resins. These 3D-printed resins with low concentrations of nanocomposites could be used as possible materials for the prevention and treatment of denture stomatitis, due to their antibiofilm activities.

## 1. Introduction

One of the most frequently used substances for fabricating complete or partial denture prostheses is poly(methyl methacrylate) (PMMA). PMMA is mainly used because of its biocompatibility, good aesthetics, ease of handling, and low cost [1]. However, PMMA has some disadvantages, such as polymerization shrinkage, low strength, low wear resistance, and, most importantly, high vulnerability to microbial and fungal colonization [2,3].

Constantly evolving technology has made dentistry one of the most advanced fields of medicine, and modern technologies are widely used in the dental field. One of the most important digital technologies to have been introduced to dentistry is computer-aided design–computer-aided manufacturing (CAD–CAM) systems [4]. They enable faster treatment with better accuracy and the ability to simulate the final result and show it to the patient in advance [4]. CAD–CAM can be used to fabricate removable dental prostheses, either using a subtractive method (milling) or an additive method, such as three-dimensional (3D) printing [4]. Three-dimensional printing is a relatively recent technology that is used to fabricate prostheses layer-by-layer [5]. Dental prostheses with undercuts, voids, complex internal geometrical elements, and anatomical landmarks can be created using 3D printing technology, which also removes operator and procedural errors [5]. Three-dimensional printing technology provides the potential to perform more effective procedures compared to conventional techniques, for complex dental operations [5].

Although 3D printing systems are popular in the dental field, they have several drawbacks, due to technical constraints [6,7]. These disadvantages are related to the mechanical and physical properties of 3D-printed materials in comparison with heat-polymerized resin [6,7]. One study found that 3D-printed resin showed an inferior double bond conversion in comparison with conventional heat-polymerized PMMA [6]. Other recent studies have shown that conventional PMMA has higher modulus of elasticity, flexural modulus, impact strength, and hardness values compared to 3D-printed resin. Moreover, 3D-printed resin displayed an inferior surface roughness compared to conventional heat-polymerized PMMA resin [7]. The clinical success and longevity of denture base materials are determined by a variety of physical properties such as surface hardness and roughness [8]. Previous studies have shown that if the surface roughness is high, there is a greater risk of bacterial and fungal adhesion and plaque accumulation, which affect the longevity of the prosthesis [9,10]. Therefore, it is important to assess the surface characteristics of denture base materials [10].

When the host is affected by local and systemic factors, the distribution or count of oral flora may change, leading to *Candida* becoming virulent. *Candida* is known to cause an oral disease known as oral candidiasis [11,12]. It has been demonstrated that the presence of a denture is a contributing factor in initiating pathology related to *Candida* [12]. Studies have shown that *Candida* is capable of adhering to the surfaces of acrylic resins of removable dental prostheses [12]. Adhesion to acrylic resin surfaces may lead to the development of infection. This could ultimately lead to varying degrees of denture-induced stomatitis, which affects 70% of denture wearers [13,14]. *Candida* adheres to the PMMA denture base directly or through a layer of denture plaque [15].

It is well established that a variety of factors play a role in the adhesion of *Candida* to the acrylic resin surface. There is evidence that certain substrate surface characteristics can influence how quickly microbes adhere to surfaces, namely the surface charge, surface free energy, hydrophobicity, and roughness [15]. Several studies [16,17] reported an increased yeast count for rougher surfaces, while others concluded that the surface topography had no effect on *C. albicans* count and adherence [18,19]. Microbial adhesion is influenced by the surface charge and the hydrophobicity of the microbial cell surface, as well as the biomaterial’s surface and structural composition [15].

Nanoparticles were introduced into dentistry with the aim of developing better material properties. Zirconium dioxide was suggested, to improve mechanical and antimicrobial properties [20]. Zirconium dioxide nanoparticles (ZrO_2_ NPs) have remarkable antimicrobial and antifungal properties, and multiple studies have investigated the antifungal and antimicrobial effects of ZrO_2_ NPs on *C. albicans* [21]. The active oxygen species produced by ZrO_2_ NPs may contribute to their improved antibacterial activity, as suggested by Gowri et al. [21]. A study by Antony et al. indicated that using ZrO_2_ NPs at concentrations of 2.5% and 5% reduced the porosity of the denture base surface and reduced the number of adherent *C. albicans*. Nevertheless, the same study concluded that 2.5% and 5% ZrO_2_ NPs had a fungistatic effect [22]. Jangra et al. found that ZrO_2_ NPs mainly targeted *Escherichia coli* and *Staphylococcus aureus* bacterial strains and *Aspergillus niger* fungal strains. ZrO_2_ NPs have shown activity against *Escherichia coli* [23]. The effect of ZrO_2_ NPs against fungal strains arises from their high surface area, and they can also inhibit fungal strain growth by disturbing cell function [23]. Previous studies [24] have examined the effects of ZrO_2_ NPs in different concentrations (0.5% 1%, 3%, and 5%) on the properties of 3D-printed denture bases, such as impact strength, elastic modulus, hardness, and surface roughness. The elastic modulus and impact strength were highest at 1% concentration and decreased gradually as the concentration was increased to 3% and 5%. Adding ZrO_2_ NPs was also observed to increase hardness [24]. Another study examined the effect of ZrO_2_ NPs on denture translucency, and showed that adding ZrO_2_ NPs, especially in low concentrations, did not affect the translucency. Thus, the study highly recommended the addition of ZrO_2_ NPs to denture bases [25].

To the best of the authors’ knowledge, no prior studies have examined the effect of adding different concentrations of ZrO_2_ NPs on the antibiofilm activities of 3D-printed denture base resins. Therefore, this in vitro study investigated the antibiofilm activities and surface roughness of 3D-printed denture base resins modified with different concentrations of ZrO_2_ NPs. The null hypothesis was that adding ZrO_2_ NPs to the 3D-printed resin would not affect the antibiofilm activity and surface roughness of the 3D-printed nanocomposite.

## 2. Materials and Methods

**Figure 1:**
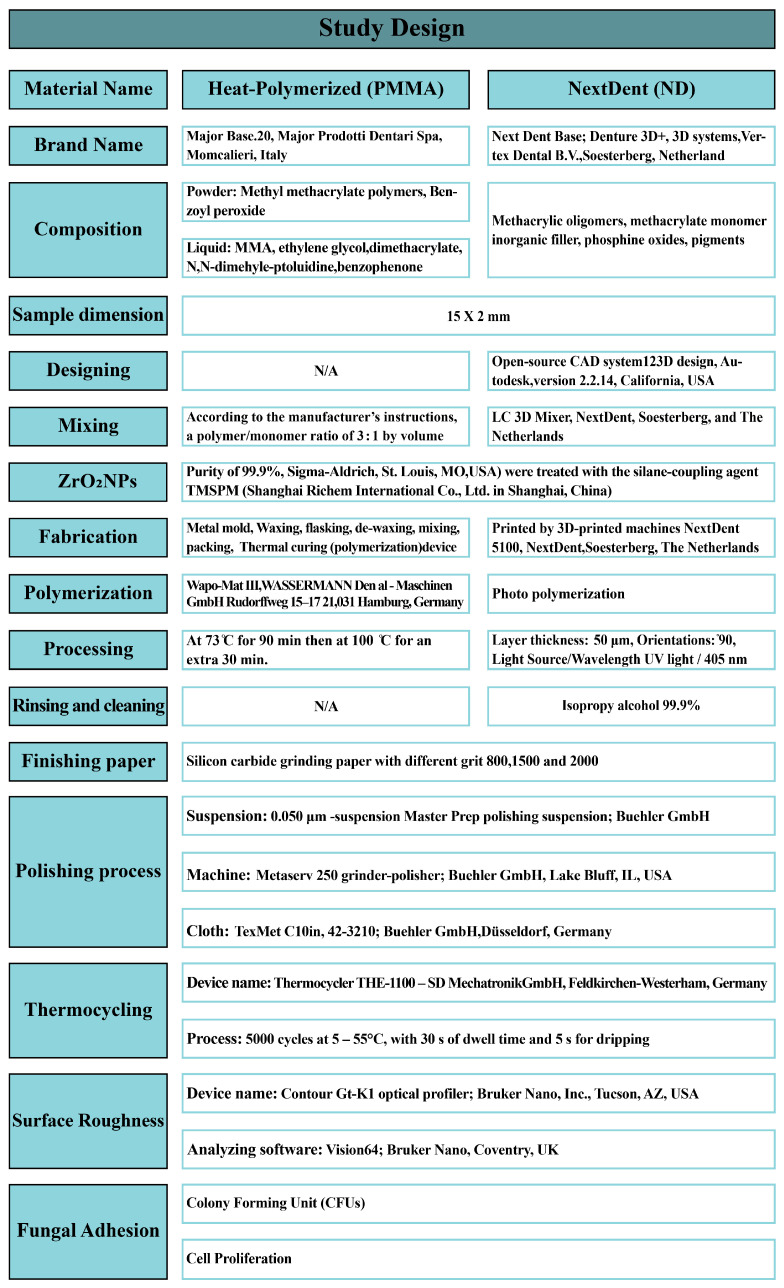
Study design and flowchart.

The in vitro samples were counted using power analysis. A study power of 80%, a significance level of 5%, and a margin of error of 5% were established using World Health Organization formulas. Six groups (n = 10), comprising one heat-polymerized resin (PMMA) group and five 3D-printed resin (NextDent) groups, were formed for a total of 60 specimens. A summary of the specimen specifications and the processing steps is presented in Figure 1.

### 2.1. Heat-Polymerised PMMA Specimen Preparation

A disc-shaped metal mold was used to create the specific dimensions of the wax pattern. The wax was invested in stone in flasks, followed by dewaxing, resulting in molds for acrylic resin packing. Then, the powder and liquid were mixed according to the manufacturer’s instructions. After packing the PMMA specimens, the flasks were placed in a thermal polymerization unit for curing. The specimen dimension specifications and processing steps are presented in Figure 1 [26].

### 2.2. Nanocomposite Mixture Preparation

ZrO_2_ NPs were added to the 3D-printed resin in various concentrations (0.5 wt%, 1 wt%, 3 wt%, 5 wt%), while one 3D-printed resin group remained without modification [27]. The ZrO_2_ NPs had an average granularity size of 40 nm and a surface area of 9 m^2^/g, based on previous TEM and SEM analyses [28,29]. The bonding between the ZrO_2_ NPs and the resin matrix was enhanced by a silane coupling agent, 3-(trimethoxysilyl)propyl methacrylate (Shanghai Richem International Co., Ltd., Shanghai, China). This was used to treat the surface of the ZrO_2_ NPs by creating reactive groups through a silanization process. The silane coupling agent was first dissolved in acetone, then the ZrO_2_ NPs were added, and the mixture was stirred for 60 min. Then, the acetone was eliminated using a rotary evaporator, followed by a cooling process to obtain the silanized ZrO_2_ NPs [30]. The silanized ZrO_2_ NPs were weighed using an electronic scale, then they were added to the 3D-printing resin. The ZrO_2_ NP-containing fluid resins were thoroughly combined and stirred using a magnetic stirrer for 30 min [31].

### 2.3. D-Printed Specimen Preparation

An open-source CAD system was used to design the disc diameter. The design was saved as an STL file and printed by a 3D-printing machine. The machine’s specifications are outlined in detail in Figure 1. The pure resin was placed on a mixing machine and mixed for half an hour, before adding the nanoparticles. The nanoparticles were added and distributed between several bottles, each containing a different concentration of nanoparticles. All the bottles were remixed before the printing process. The layer thickness and degree of orientation are presented in detail in Figure 1 [32]. Subsequently, the specimens were exposed to UV light for polymerization. Then each specimen was wiped using isopropyl alcohol and immersed in a bowl containing glycerol during the post-curing process. The post-curing process had a duration of 10 min in a post-curing unit, according to the manufacturer’s guidance. The machine’s specifications are described in detail in Figure 1 [33].

Excess resin supporting the 3D-printed resin specimens was trimmed using slow-speed rotary instruments. Finishing was carried out using silicon carbide paper (500 and 800 grit). All samples were polished using a 0.050 µm suspension, combined with a polishing cloth in wet conditions [34]. The finishing and polishing processes are further explained in Figure 1. To confirm that the polishing instruments were applied to the specimens with an equal amount of pressure, the samples were polished by one researcher. A thermocycling machine was used to simulate the changes in temperature occurring intraorally over half a year. The machine manufacturers and processing details are listed in Figure 1 [35].

### 2.4. Surface Roughness (Ra, µm)

To measure Ra, a non-contact profilometer was used. A software package was used to analyze the obtained images. Three different points were scanned for each specimen with 0.01 mm resolution. Finally, the averaged surface roughness (µm) for each specimen was calculated using the captured images. The machine manufacturers are listed in Figure 1.

### 2.5. Fungal Adhesion Assay

The fungal adhesion assay experiment was performed as reported by Murat et al. [36]. Briefly, a reference strain of *C. albicans* (ATCC 10231) was cultured on Sabouraud dextrose agar (SDA) at 30 °C for 48 h, to prepare the adjusted inoculum [36]. All specimens were sterilized with 70% isopropyl alcohol (IPA), then placed in separate 12-well sterile microplates with 2 mL of the fungus suspension and incubated for two days at 37 °C. To remove non-adherent cells, the specimens were rinsed twice with phosphate-buffered saline (PBS). To dislodge the adhered cells, all the specimens were transferred to new microplates and 2 mL of PBS was added to each well. Finally, the plates were placed in an ultrasonic machine for 5 min [17].

#### 2.5.1. Colony Forming Unit Assay

A direct culture method using a colony-forming unit (CFU) assay was performed to count the adherent cells by streaking an appropriately diluted suspension on agar media and incubating for 24 h at 37 °C [36]. The yeast colonies were counted using a counting marker (colony counter; SP Scienceware, Bel-Art Products). The experiments were performed in triplicate and repeated three times, with similar results.

#### 2.5.2. Cell Proliferation Assay

A cell counting kit (CCK-8/WST-8) (ab228554, Abcam, Waltham, MA, USA) based on monosodium salt WST-8 was used to count the number of viable cells in accordance with the manufacturer’s instructions Each sample was transferred in a volume of 100 mL to a 96-well plate, and 10 mL of the dye was then applied to each well. After 3 h of incubation at 37 °C in the dark, cellular dehydrogenases degraded WST-8 to an orange formazan product that was soluble in the buffer. The WST-8 kit is a colorimetric assay in which salt is reduced by metabolically active cells, to form an orange formazan product that is quantifiable by plate reader spectrophotometry. It is a direct assay for cell viability and proliferation, indicated by the color intensity. The absorbance of the cell suspension was determined using a microplate reader at 450 nm (Bio-Rad xMark^TM^ Microplate Spectrophotometer; Hercules, CA, USA). The quantity of formazan produced was directly correlated with the number of live cells [37].

### 2.6. Statistical Analysis

A Shapiro–Wilk test was used to examine the normality of the data. A lack of normality in the data is indicated by insignificant *p*-values. Hence, non-parametric tests were used for inferential data analysis. The significance of the mean variation due to the variation in concentration levels was investigated using a Kruskal–Wallis test. Furthermore, a pairwise comparison was made using a Mann–Whitney *U* test. *p*-values less than 0.05 were considered statistically significant. GraphPad Prism 8 software (GraphPad Software, Inc., La Jolla, CA, USA) was used for graphing.

## 3. Results

**Figure 2:**
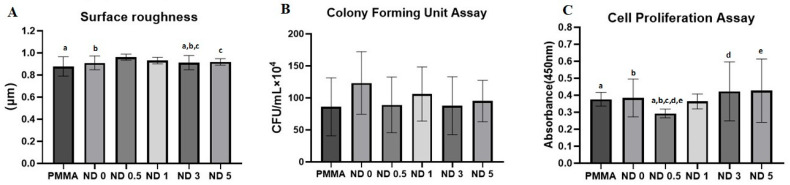
Results of surface roughness (**A**) and biofilm assay testing (**B**,**C**). Identical lower-case letters indicate significant differences in pairwise comparisons.

Table 1 and Figure 2A summarize the results of the roughness, mean, and standard deviation (SD) for the control group and different concentrations of ZrO_2_ NPs. The overall variation in the mean was found to be statistically significant (*p* = 0.032). Hence, a post hoc test was performed for pairwise comparison. In comparison to the control group, no significant difference in Ra was found for all the 3D-printed groups,, except the 3% group, which showed a significant increase in Ra with a *p*-value of less than 0.025. No significant differences were observed between the 3D-printed resin groups, except for 0% vs. 3% and 3% vs. 5% ZrO_2_ NPs, with a *p*-value of less than 0.025. The maximum Ra value was found at the 3% concentration (0.96 ± 0.06 µm), while the minimum value was found at concentrations of 0.5% and 5% (0.88 ± 0.087 µm).

**Figure 3:**
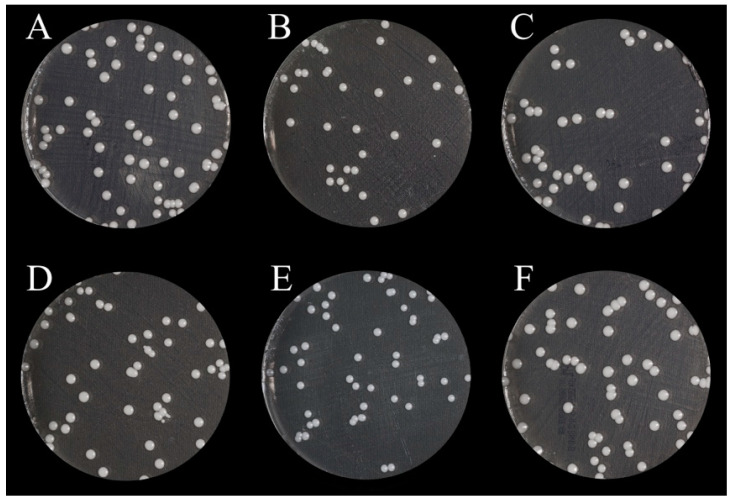
CFU images of the tested groups, showing the growth of adherent *C. albicans* in each group: (**A**) heat-polymerized PMMA, control group; (**B**) NextDent group with 0% ZrO_2_ NPs; (**C**) NextDent group with 0.5% ZrO_2_ NPs; (**D**) NextDent group with 1% ZrO_2_ NPs; (**E**) NextDent group with 3% ZrO_2_ NPs; (**F**) NextDent group with 5% ZrO_2_ NPs.

Table 2 and Figure 2A summarize the results of the significance, mean, and SD for CFUs for the control group and different concentrations of ZrO_2_ NPs. The highest *Candida* count was found at the 0% concentration (123.3 ± 48.9), while the lowest count was found in the control group (0.86 ± 45.31). The overall variation in *Candida* count was found to be statistically insignificant (*p* = 0.75). Figure 3 shows representative photographs of the results of the CFUs, demonstrating the effects of ZrO_2_ NPs against the growth of adherent *C. albicans* in the different groups.

Table 3 and Figure 2C summarize the results of the significance, mean, and SD for the cell proliferation assay, for the control group, and with different concentrations of ZrO_2_ NPs. The overall variation in the mean was found to be statistically significant (*p* = 0.006). Hence, a post hoc test was performed for pairwise comparison. In comparison to the control group, a significant decrease in the mean was observed at 0.5% concentration, with a *p*-value of less than 0.025, while there was no significant difference between the other 3D-printed groups. Among the 3D-printed resins, the addition of 0.5% ZrO_2_ NPs resulted in a significant decrease in the mean compared to the other groups (0.5% vs. 0%, 0.5% vs. 1%, 0.5% vs. 3%, and 0.5% vs. 5%), with a *p*-value of less than 0.025. The highest mean for the cell proliferation assay was found at a concentration level of 5%, while the lowest mean was found at the 0.5% concentration.

## 4. Discussion

In the present study, the addition of ZrO_2_ NPs to 3D-printed resin affected the surface roughness, particularly at high concentrations. Additionally, in terms of antibiofilm effectiveness, cell proliferation assays revealed significant differences between the tested groups. Therefore, the null hypothesis was rejected.

It has been found that the presence of a denture in the oral cavity acts as a medium in the development of denture stomatitis [11,12]. Therefore, ZrO_2_ NPs were selected as a potential solution to this problem. ZrO_2_ NPs possess antimicrobial and antifungal properties, mainly against *Escherichia coli*, *Staphylococcus aureus*, and *Aspergillus niger* [21]. Their mechanism involves the production of active oxygen species, which inhibit the normal budding process of pore formation in the cell wall. This causes the cell wall to disintegrate via ion outflow, which inhibits fungal growth by disturbing cell function [21,22,23]. A denture in the oral cavity is constantly subjected to thermal stresses. Therefore, the specimens were subjected to 5000 thermal cycles, to simulate the changes occurring over half a year of clinical use in the oral cavity [35].

Rough denture bases are easily stained and serve as a breeding ground for bacteria that cause denture stomatitis [38]. Additionally, a rough surface protects bacteria from shearing pressures during denture cleaning, making it challenging to remove the bacteria [15]. Surface roughness is influenced by multiple factors, including handling and polishing, the type of resin used, the coupling agent used to dissolve polymer chains, monomer elution, filler shape, and filler concentration [13,39]. In the current study, heat-polymerized PMMA displayed a lower surface roughness compared to all the 3D-printed resins. The layer-by-layer printing method might have been the reason for the elevated Ra. Additionally, the specimens’ surfaces had more compact stepwise edges due to the printing orientation (90°), which made the surface between layers rougher [7,24,40]. These findings are in line with those of Alshaikh [24], who reported that heat-polymerized PMMA showed a lower surface roughness compared to 3D-printed resins. According to this study, the addition of ZrO_2_ NPs to 3D-printed resin resulted in a significant increase in Ra. These findings are in agreement with those of previous studies, which reported that adding ZrO_2_ NPs to resin specimens caused a significant increase in roughness in comparison to the control material [11,41].

This study showed variations in *C. albicans* adhesion with the addition of ZrO_2_ NPs. In the current study, a cell proliferation assay revealed that the 0.5% ZrO_2_ NPs 3D-printed resin group significantly reduced the *C. albicans* count when compared to heat-polymerized PMMA and the other 3D-printed resin groups. This may be due to the antibacterial effect of ZrO_2_ NPs, which has been described in numerous studies [21,23]. The decrease in the *C. albicans* count could be attributed to the presence of specific ZrO_2_ NPs on the specimen surface in close proximity to the *C. albicans* cell membrane, which could impart antifungal capabilities to the modified resin [42]. Nanoparticles can come into contact with one another as they pass through the fungal cell membrane and interfere with the metabolic process through a variety of mechanisms, such as hydrophobic contact, electrostatic attraction, and van der Waals forces. This alters the shape and function of the cell membrane and inhibits the normal budding process [42,43]. Studies have explained the mechanism of the antimicrobial effect in this material, in which nanoparticles can wrap around the microbial cells, thereby inhibiting the normal budding process of the cells. Moreover, the antimicrobial effect could have been due to the disintegration of the microbial cell via the formation of pores, which cause ion outflow and changes in the cell structure, leading to cell death [44]. Nanoparticles have the potential to create reactive oxygen species. Another mechanism involves the interaction of reactive oxygen species with cell membranes to increase cell permeability, causing the leakage of intracellular contents and ultimately cell death [45]. Based on this finding of antibiofilm activities at low concentrations, it was predicted that high concentrations would confer a greater effect, due to the increased density of ZrO_2_ NPs on the specimens’ surface. However, higher concentrations were correlated with increased cell proliferation, which may be attributed to the agglomeration of ZrO_2_ NPs and formation of clusters within the resin matrix or at the resin surface. On the other hand, the CFU results showed an insignificant decrease in *C. albicans* adhesion with the addition of ZrO_2_ NPs, which is in agreement with a previous study [11].

In a previous study, Hamed et al. [13] investigated the effect of artificial ageing on the antifungal activities of ZrO_2_ NPs. They found that a conventional PMMA denture base modified with ZrO_2_ NPs showed concentration-dependent antifungal effects. The mechanism of action was attributed to the direct contact of ZrO_2_ NPs on the surface of the specimen with the cell surface. Additionally, the Ra increased with the addition of ZrO_2_ NPs but *C. albicans* adhesion decreased, confirming the antifungal properties of the modified surface. Hamed et al. hypothesized that ions leach out similarly to other NPs (such as Ag NPs) using a disc diffusion method and a lack of clear zones confirmed the absence of this mechanism for ZrO_2_ NPs. Fouda et al. [14] added nanodiamonds (NDs) to denture base resins and reported an antifungal effect, even with an increased Ra at high concentrations of NDs [14]. This variation in results can be attributed to the resin type, since Hamed et al. and Fouda et al. added ZrO_2_ NPs and NDs to conventional PMMA denture base resin, respectively, while in the present study, an ester-based 3D-printed resin was used. Therefore, the results should be compared with caution.

The formation of clusters is expected as the concentration of NPs increases. The effect of the NPs was mainly attributed to their nano-size and the increased surface area [41]. When NPs agglomerate, the clustered nanoparticles have a lower surface area compared to separate NPs. The agglomeration of NPs masked the activity of the core of the cluster, while only the surfaces at the cluster boundaries demonstrated antifungal activity. No greater effect on *C. albicans* adhesion was observed for increased concentrations [46]. This is in agreement with the results reported by Fouda et al., who analyzed the surface of ND-modified denture base resins using a scanning electron microscope and found that low ND concentrations improved the surface properties, while increased ND concentrations resulted in the formation of clusters [14]. The researchers attributed this effect to the saturation of inter-polymeric chains of the resin matrix with NDs. Particles added after saturation were collected to form clusters.

Among factors that could affect the surface properties are resin type and hydrophobicity, which are directly related to *C. albicans* adhesion [47]. Hydrophobic materials have been reported to be more prone to *C. albicans* adhesion through hydrophobic interactions between the denture surface and the hydrophobic cell surface [48]. Two previous studies [49,50] compared the hydrophobicity of 3D-printed resin with that of conventional PMMA. Freitas et al. [49] found that 3D-printed resin showed a lower contact angle (indicating greater hydrophilicity) when compared with conventional PMMA acrylic. However, 3D-printed resins showed the highest *C. albicans* adhesion among the tested resins, which was mainly attributed to their surface roughness. Meirowitz et al. [50] compared the correlation of *C. albicans* adhesion to PMMA and 3D-printed resins with surface roughness and hydrophobicity. They found that 3D-printed resins showed an increased *C. albicans* adhesion, which was associated with an increased contact angle (greater hydrophobicity), while surface roughness was not a contributing factor. The variations in results between these two studies necessitate further investigations of different 3D-printed resins, in terms of hydrophobicity and its effect on surface properties. Further investigations are required to elucidate the mechanisms of action of nanocomposites at different concentrations against biofilm formation, and to detect the efficiency of incorporation of NPs inside cells.

Methods based on the capacity of yeast cells to expand in either solid or liquid media are utilized in cell viability assessment. Analysis of the number of colonies is one of the techniques that is most frequently utilized for colony-forming units [51]. The nature of the resulting biofilms is substantially influenced by the initial cell concentration of a biofilm assay [36]. CFU assays are simple and affordable; however, a drawback is the lengthy waiting period for results. More crucially, despite the ability of these tests to estimate the level of growth inhibition, they are unable to estimate live yeast cells or cells that are unable to reproduce [37]. Methods that depend on staining with dyes appear to produce more accurate results regarding the death rate of cells. One method that employs dye staining is cell counting kits (CCK-8/WST-8), which was the method used to measure *C. albicans* adhesion in our study. According to previous studies, a cell proliferation assay using CCK-8/WST-8 is the most reliable, accurate, and practical technique for determining the amount of biofilm for the studied *C. albicans* strains [52].

Surface roughness is an important factor related to *C. albicans* adhesion, as reported in previous literature reports [16,17]. However, the results of our study demonstrated that there was no significant effect of the surface roughness on the colony-forming unit assay, or on the cell proliferation assay. This is in line with recent studies, as it was found that there was no significant relationship between surface roughness and *C. albicans* adhesion to denture base resins [18,19]. For evaluation of the surface roughness, only the average roughness (Ra) was investigated. No additional parameters, such as the mean roughness depth (Rz), the maximum peak height (Rp), or the distance between peaks, were measured. Including these parameters in future studies along with Ra may produce variations in the results and their correlation with microbial adhesion. Regardless of the effect of surface roughness, cell proliferation assays showed a significant decrease in the *C. albicans* count for the 0.5% ZrO_2_ NPs 3D-printed resin group compared with PMMA and all other 3D-printed resin groups. This proves that ZrO_2_ NPs had an antifungal effect.

ZrO_2_ NPs were added to different denture base resins to improve the strength of the modified resin [24], and the superior strength of the nanocomposites was reported in comparison to the pure resins. Recently, Alshaikh et al. and Khattar et al. [25] recommended adding ZrO_2_ NPs to 3D-printed resins. They reported that the strength increased with high concentrations, while low concentrations were recommended in terms of optical properties. In terms of antibiofilm activities, a low concentration (0.5%) can be recommended with caution, until further investigations prove the effect of ZrO_2_ NPs on different 3D-printed resins. The clinical implications of this study include the possibility of adding ZrO_2_ NPs into denture base resin, to potentially reduce and inhibit *Candida* adherence and multiplication. Thus, in vivo research is required to examine the impact of these variables and the longevity of the effect of ZrO_2_ NPs on 3D-printed resins. Furthermore, the strength of these ZrO_2_ NPs is crucial, because it may enable complete denture wearers with limited manual dexterity or cognitive impairments to maintain better denture care.

A limitation of this study is that the specimens were tested under conditions that differed from those in the oral cavity environment, as the oral cavity environment is constantly changing. The pH, the presence of certain microbes, and saliva can all have an impact on the adherence of bacteria to denture surfaces [53]. The specimens in this study did not replicate the design of dentures and they were not subjected to all the relevant intraoral conditions, such as changes in pH values; contact with saliva, beverages, and food; and denture cleaning. Only one type of NP and one printing orientation were employed. Additionally, dynamic loading was not present, and the heat cycling ageing only accurately represented six months of intraoral use. Different antifungal agents [54] and nanoparticles at different concentrations, with different printing orientations [55] should be investigated. This report also suggests the use of various additional 3D-printed materials assembled in a denture configuration and subjected to mechanical and thermal stresses that are similar to those found in the intraoral cavity.

## 5. Conclusions

With advances in digital technology for denture fabrication, researchers have focused on the improvement of 3D-printed resins in terms of their mechanical and biological properties through modification with nanoparticles. Due to the ability of ZrO_2_ NPs to improve the mechanical performances of 3D-printed resins, further investigation is recommended. As no studies have investigated the antimicrobial activities of 3D-printed resins modified with ZrO_2_ NPs, this study focused on evaluating the enhanced properties of 3D-printed nanocomposite resins compared to pure resins. Denture base fabrication using this nanocomposite is considered an effective therapeutic method to inhibit microbial adhesion and proliferation, particularly in elderly patients wearing complete dentures. The surface roughness of the 3D-printed resin is comparable to that of the conventional heat-polymerized PMMA denture base resin. The addition of ZrO_2_ NPs at low concentrations did not affect the Ra of the 3D-printed resin. PMMA and the pure 3D-printed resin showed no differences in *C. albicans* adhesion. The group with the addition of a low concentration of ZrO_2_ NPs (0.5%) showed a significantly lower level of *C. albicans* adhesion compared to the other groups. Future clinical trials are required to evaluate and confirm the impact of these nanocomposites on oral biofilm activity under natural oral conditions.

## Figures and Tables

**Table 1:** Mean, SD, and significance of surface roughness (µm).

Material	ZrO_2_ NPs%	Mean (SD)	*p*-Value
HP	Control	0.88 (0.087) ^a^	0.032 *
NextDent	0%	0.92 (0.06) ^b^	
	0.5%	0.91 (0.03)	
	1%	0.93 (0.03)	
	3%	0.96 (0.06) ^a,b,c^	
	5%	0.91 (0.03) ^c^	

* Statistically significant at 0.05 level of significance. Identical lower-case letters indicate significant differences between the means.

**Table 2:** Mean, SD, and significance of CFUs.

Material	ZrO_2_ NPs%	Mean (SD) CFU/mL × 10^4^	*p*-Value
HP	Control	86 (45.31)	0.52 *
NextDent	0%	123.3 (48.9)	
	0.5%	89.13 (43.44)	
	1%	106.1 (42.31)	
	3%	87.75 (45.16)	
	5%	95.13 (32.31)	

* Statistically significant at a 0.05 level of significance.

**Table 3:** Mean, SD, and significance of cell proliferation assay.

Material	ZrO_2_ NPs%	Mean (SD)	*p*-Value
HP	Control	0.38 (0.04) ^a^	0.006 *
NextDent	0%	0.39 (0.11) ^b^	
	0.5%	0.29 (0.03) ^a,b,c,d,e^	
	1%	0.36 (0.43) ^c^	
	3%	0.42 (0.17) ^d^	
	5%	0.43 (0.19) ^e^	

* Statistically significant at a 0.05 level of significance. Identical lower-case letters indicate significant differences between the means.

## Data Availability

Not applicable.
